# Adipose Tissue and Adipokines: The Association with and Application of Adipokines in Obesity

**DOI:** 10.1155/2014/328592

**Published:** 2014-09-17

**Authors:** Muhammad Khan, Frank Joseph

**Affiliations:** Countess of Chester Hospital NHS Foundation Trust, Countess of Chester Health Park, Liverpool Road, Chester CH2 1UL, UK

## Abstract

2014 marks the 20th anniversary of adipokines. Through the identification of leptin, our perceived understanding of adipose tissue was changed instantaneously. From a simple dormant site of energy storage, adipose tissue is now recognized as an integral hub of various hormones known as adipokines. Although great strides have been made in characterizing these hormones in health, research also shows they are significantly implicated in a series of pathologies. One such condition is obesity. Defined as an excess of adipose tissue, obesity remains one of the greatest healthcare epidemics of the 21st century. With no definitive treatment, attention has shifted to understanding the role of adipokines in obesity. This review provides an introduction to the salient obesity-related adipokines and their possible application as a treatment for obesity.

## 1. Introduction

Defined as a body mass index (BMI) ≥ 30 kg/m^2^, obesity reflects the excessive enlargement of adipose tissue [1]. Over the last decade we have realized the ramifications posed by obesity, yet the worldwide burden due to obesity is said to have doubled since the 1980s [2]. Moreover, obesity increases the risk of several debilitating comorbidities which includes diabetes, cardiovascular disease, and several forms of cancer [1]. With no means of effectively curbing the increasing tide of obesity, this inexorable rise in mortality and morbidity is set to overwhelm healthcare systems [1]. Consequently, it is evident why obesity is hailed as one of the leading healthcare epidemics of the 21st century [2].

Realizing the gravity of the situation, attention has shifted to understanding the intricate mechanisms dictating obesity. Although other reviews have discussed the multifactorial components underpinning obesity [3], one element gaining momentum is the role of the adipose tissue itself. Adipose tissue is categorically divided into white adipose tissue (WAT) and brown adipose tissue (BAT). Although the latter orchestrates heat production and energy expenditure, the former since antiquity has been deemed as being metabolically inactive. For decades, the established dogma concerning WAT was that it served as the primary location of triglyceride storage and fatty acid release [4]. However, following the seminal paper in 1994 characterizing the hormone leptin from WAT [5], there was a significant paradigm shift in how we perceived WAT. What was initially labelled a mere dormant tissue, WAT, was now recognized to be an integral endocrine organ which secretes a plethora of biologically active polypeptides known as adipokines [4].

## 2. Adipokines

Adipokines refer to proteins secreted from adipose tissue [4]. Although their existence was confirmed in 1994 [5], the concept of adipokines has been postulated since 1948. In contrast to the status quo, both Shapiro and Wertheimer felt that adipose tissue not only was metabolically active but also additionally regulated energy expenditure through both “nervous and endocrine factors” [6]. They had unknowingly foreseen the role of WAT. Structurally WAT is mosaic in nature with a third consisting of adipocytes and the remainder being a mesh of nervous tissue, immune cells, connective tissue matrix, and stromovascular cells [7]. Although adipocytes are responsible for expressing a myriad of adipokines, the majority of adipokines are derived from the other components of WAT [4]. Nevertheless, each element within WAT works in tandem, thus making WAT a functional endocrine unit.

**Figure 1:**
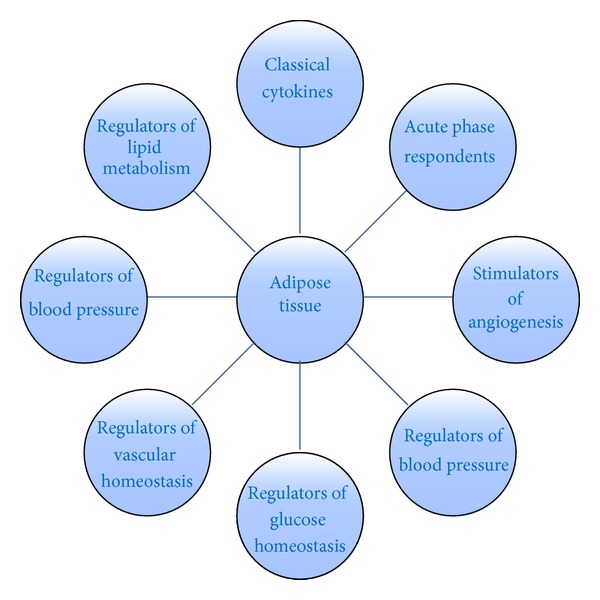
Overview of the various physiological functions of adipokines expressed and secreted from adipose tissue [9].

Adipokines are known to act both locally and systemically and express a variety of receptors, thereby allowing it to respond to signals from other hormonal networks but also the central nervous system (CNS) [4]. With the capacity to both direct efferent signals and respond to afferent signals, adipose tissue forms a vast highway of cross-communication between numerous organs within the body. It is this expansive network that results in the diverse physiological roles of adipokines (Figure 1), yet the importance of adipokines can be emphasized further by the adverse metabolic consequences arising in obesity [8]. In the obese state, the excess growth in adipose tissue has been shown to alter the adipokine profile, thereby initiating a detrimental cascade of metabolic disturbances [9]. This change is said to be influential in the development of hyperglycemia, insulin resistance, dyslipidemia, and an increased risk of cardiovascular diseases seen typically in obesity [8]. This review aims to delineate the profile of several notable adipokines implicated in obesity and obesity-related metabolic disorders, which includes leptin, adiponectin, tumor necrosis factor-alpha (TNF-*α*), interleukin-6 (IL-6), plasminogen activator inhibitor-1, visfatin, and chemerin, whilst exploring their therapeutic role against obesity.

## 3. Leptin

Prior to its identification, parabiosis studies involving mice expressing the recessive obese (*ob*) and diabetes (*db*) genes suggested the existence of a hormone capable of relaying information regarding energy expenditure between the periphery and the CNS [10]. This hormone was leptin. Derived from the Greek word* leptos*, meaning thin, leptin is a 16-kDa polypeptide product of the* ob* gene which bears structural homology to cytokines [9]. Leptin is predominantly expressed by adipocytes in subcutaneous (SC) WAT in a manner proportional to adipose tissue mass and nutritional status [7].

Leptin primarily regulates energy homeostasis by controlling satiety and body weight through three leptin-sensitive neurons found within the arcuate nucleus of the hypothalamus: neuropeptide Y (NPY), *γ*-aminobutyric acid (GABA), and proopiomelanocortin (POMC) neurons [11, 12]. Following its release, leptin crosses the blood-brain-barrier (BBB) and inhibits NPY and GABA neurons, whilst simultaneously stimulating POMC neurons. As both NPY and GABA are classed as orexigenic neurons, whereas POMC is deemed an anorexigenic neuron, leptin promotes the sensation of satiety and increases energy expenditure [13]. Although leptin predominantly acts on the hypothalamus, leptin also affects adipose tissue. Studies have shown the autocrine properties of leptin through leptin mediated alteration of adipocytes within WAT into those rich in mitochondria capable of oxidizing stored triglycerides, thereby increasing energy expenditure [14].

The novel ability of leptin to regulate food intake and body weight sparked mass commotion. Could such results be achieved through exogenously administering leptin in obese humans? Was leptin the obesity panacea? For those with congenital leptin deficiency, a condition marred by extremely low levels of circulating leptin, extreme obesity, and severe hyperphagia, daily SC injections of leptin completely reversed the aforementioned phenotype [15]. Although congenital leptin deficiency can cause obesity, it only accounts for a minute subset of cases. Rather the most common cause of obesity is a chronic state of energy abundance [3]. In these individuals, exogenous leptin replacement therapy only provided substantial weight loss in a significantly small subset of patients and, additionally, the higher doses required to trigger weight loss were shown to induce swelling and skin irritation at the site of injection [16]. These findings proved that leptin monotherapy failed to produce clinically relevant results applicable to the wider obese population. However, new studies have emerged utilizing leptin with other agents that increase the responsiveness to leptin. One such combination includes metreleptin and pramlintide, which are synthetic forms of the hormones leptin and amylin, respectively. Although phase II trials showed promising weight loss, further studies were abruptly halted due to the adverse formation of antibodies against metreleptin [17]. It is now understood that failure of leptin in the vast majority of obesity may be due to leptin resistance [4].

Leptin resistance reflects elevated levels of circulating leptin, in addition to hyperphagia and increased adiposity [9]. This pathological state results in resistance to both endogenous and exogenous sources of leptin. Desensitization to leptin is a hallmark finding in energy abundance-related obesity [4]. Moreover, studies have shown the severity of obesity to be directly proportional to the concentration of circulating leptin [18]. Although not fully understood, leptin resistance is attributable to defects in transport of leptin across the BBB and/or leptin signaling in both target neurons and cells [4]. Additionally, increased expression of the peptides suppressor of cytokine signalling-3 (SOCS-3) and protein-tyrosine phosphatase 1B (PTP-1B) further inhibits leptin signaling [9]. The increased expression of SOCS-3 is also thought to induce a state of insulin resistance [9].

Alongside its role in energy homeostasis, leptin is able to modulate the immune system due to a combination of it bearing structural similarity to cytokines and its class I cytokine receptors being found on immune cells such as monocytes, lymphocytes, and neutrophils [19]. Therefore, it is believed that the chronic subinflammatory state observed in obesity is attributed to the elevated leptin levels through upregulation of phagocytosis by macrophages, promotion of T-helper 1 cell responses, and mediating the release of further proinflammatory cytokines such as TNF-*α* and IL-6 [19].

## 4. Adiponectin

Adiponectin is a 30-kDa polypeptide released exclusively from adipocytes of WAT [20]. With expression being greater from SC WAT compared to visceral WAT, adiponectin represents the most abundantly expressed and adipose-specific adipokine [7]. In contrast to leptin, adiponectin levels decrease prior to the onset of obesity and insulin resistance and attenuate the degree of inflammation and insulin resistance [21]. It is believed that this inverse relationship between obesity, insulin resistance, and adiponectin is crucial to their pathogenesis [21].

Adiponectin predominantly affects the liver, skeletal muscle, vascular wall, and endothelial cells [9]. Within the liver, activation of AMP protein kinase (AMPK) by adiponectin results in a series of metabolic improvements which includes enhancement of insulin sensitivity, reduction in the influx of nonesterified fatty acids (NEFAs), increase in the oxidation of fatty acids, and overall reduction in the expression of enzymes involved in hepatic gluconeogenesis [22]; NEFAs are the major components of triglycerides. Likewise, activation of AMPK in skeletal muscle also increases insulin sensitivity due to stimulation in fatty acid oxidation and glucose utilization by the skeletal muscle. Additionally, adiponectin exerts a protective role in the vascular wall through inhibition of monocyte adhesion and transformation of macrophages to foam cells by reducing the expression of adhesion molecules and scavenger receptors, respectively [23]. Within the endothelial cells, adiponectin increases the production of nitric oxide, a vasodilator, whilst concurrently stimulating the formation of new blood vessels [4]. Moreover, adiponectin has demonstrated an ability to dampen the inflammatory response of endothelial cells by inhibiting the TNF-*α* mediated activation of nuclear factor-kappaB (NF-*κ*B), a key protein complex involved in the regulation of the immune system [9].

The variety of beneficial effects mediated by adiponectin has led to adiponectin being regarded as a beneficial adipokine [21]. Researchers have strived to harness the anti-inflammatory, antiatherogenic, and antidiabetic effects of adiponectin and extrapolate them to a pharmaceutical agent that stimulates the secretion and/or action of this potent adipokine. Despite animal studies demonstrating profound and sustainable weight reduction and amelioration of insulin resistance [24], the vision of providing an adiponectin adjunct against human obesity and insulin resistance has not been transcended into clinical trials.

Nevertheless, utilization of adiponectin may prove to be influential in the treatment of obesity-related nonalcoholic steatohepatitis (NASH), a condition underpinned by inflammation and accumulation of fat and fibrous tissue in the liver [25]. Deemed as the leading form of chronic liver disease within the Western world, NASH is characterized by low levels of circulating adiponectin [25]. Subsequently, adiponectin may serve not only as a diagnostic marker for NASH [26] but also as a useful hepatoprotective treatment due to its pleiotropic ability to reduce the apoptosis of hepatocytes, subdue the degree of hepatic inflammation and fibrosis, decrease the influx of free-fatty-acids (FFAs) to the liver, and induce expression of anti-inflammatory cytokines [25].

## 5. Tumour Necrosis Factor-Alpha

Tumor necrosis factor-alpha (TNF-*α*) is a 26-kDa transmembrane protein which exerts its physiological actions following cleavage to its active 17-kDa polypeptide form [4]. Predominantly known for its role in inflammation, researchers began to speculate its possible involvement in energy homeostasis after identifying its ability to induce cachexia* in vivo* [27]. At first, scientists proposed that TNF-*α* was implicated in the pathogenesis of cachexia, yet it became apparent that TNF-*α* was positively correlated with obesity with expression in adipocytes [28].

The manner in which TNF-*α* exerts its negative metabolic effects is a complex one. One mechanism involves TNF-*α* mediated modulation of gene expression within metabolically active adipose and liver tissue [4]. Within adipose tissue, TNF-*α* dampens the genes regulating the influx and storage of glucose and NEFAs, represses genes implicated in the formation of both lipids and adipocytes, and alters the expression of adipokines such as adiponectin and IL-6 [29]. Similarly, TNF-*α* represses the genes regulating glucose utilization and metabolism at the liver, but also those involved in oxidation of FFAs. However, TNF-*α* simultaneously increases the synthesis of cholesterol and fatty acids at the liver by enhancing the expression of genes involved in both processes [29]. Another mechanism involves TNF-*α* mediated impairment of insulin signaling at both the skeletal tissue and the adipocyte level [9].

Alongside being associated with the development of obesity and insulin resistance, TNF-*α* is central to the chronic inflammatory state present in obesity [4]. Such an effect is coordinated with TNF-*α* activation of NF-*κ*B which is crucial to the transcription of numerous genes linked to the inflammatory process [9]. Furthermore, TNF-*α* has also been associated with increasing the rate of atherosclerosis, a feature commonly increased in obesity, due to its ability to induce the expression of adhesion molecules in both endothelial cells and smooth muscle cells found in the vascular wall [30].

From being considered the mediator of cachexia to a pivotal regulator of obesity and insulin resistance, the role of TNF-*α* has diversified since its identification in adipose tissue in 1993 [28]. With elevated levels of TNF-*α* being shown to cause a spectrum of negative effects, it was proposed that neutralization or deletion of genes coding for TNF-*α* receptors could possibly improve both adiposity and insulin resistance. Although such effects have been demonstrated in obese rodents [31], clinical trials utilizing such techniques in obese and/or insulin resistant humans either are lacking or have proven inconclusive. Nevertheless, with TNF-*α* playing such a significant part in the above-mentioned pathological process, its possible role as an antiobesity agent remains topical and an exciting avenue of further research.

## 6. Interleukin-6

Interleukin-6 (IL-6) is a prominent cytokine known for its ability to potentiate and modulate the immune system [32]. Therefore, scientists were left perplexed when they realized that 15–35% of circulating IL-6 in the noninflammatory state arose from adipose tissue [33]. Over the last decade it has now become apparent that the significance of this 185 amino acid protein extends beyond inflammation. With IL-6 being expressed by both adipocytes and the stromovascular matrix of visceral WAT, IL-6 is an additional adipokine implicated in the genesis of obesity and insulin resistance [7].

Studies have shown that the secretion and expression of IL-6 are directly proportional to the degree of obesity, glucose intolerance, and insulin resistance [34]. With levels of circulating IL-6 increasing with weight gain and decreasing upon weight loss, assessment of genetic variations in the IL-6 gene has shown that it is linked to both indices of obesity and perturbations in the homeostatic regulation of glucose and insulin [35]. Albeit not fully comprehended, the mechanism for IL-6's effects depends upon whether IL-6 is acting on peripheral tissue or in the CNS.

Peripherally, IL-6 impairs insulin signaling overall by dampening the expression of insulin receptor substrate 1 (IRS-1), thereby leading to inhibition of the insulin signaling cascade [34]. Furthermore, IL-6 also induces the expression of SOCS-3, thus inhibiting insulin signaling [9]. Additionally, IL-6 represses the expression of adiponectin which negates any beneficial effects of this adipokine [4].

Centrally, levels of IL-6 have been shown to be decreased and there is a negative correlation between body weight and levels of central IL-6 [36]. Indeed, the central deficiency in IL-6 may result from a possible underexposure of IL-6 to regions within the brain known to regulate body weight [32]. Such discrepancies between the effects of IL-6 both peripherally and centrally can be demonstrated by the differences arising following infusion of IL-6 at these sites. IL-6 administered peripherally results in elevated levels of triglycerides and glucose, whilst concurrently increasing the degree of insulin resistance. However, delivery of IL-6 centrally leads to an increase in energy expenditure [36].

IL-6 is a novel adipokine which influences the development and evolution of obesity and insulin resistance [36]. Although central administration of IL-6 leads to increased energy expenditure and reduced adiposity [32], it is yet to be shown whether such positive effects arise in clinical trials on obese humans. This may be due to the risk of IL-6 exposure increasing the likelihood of myelomas as IL-6 is responsible for the differentiation and proliferation of immune cells [37].

## 7. Plasminogen Activator Inhibitor-1

Plasminogen activator inhibitor-1 (PAI-1) is a 45-kDa glycoprotein that is primarily expressed by hepatocytes and endothelial cells [9]. By acting as an antagonist for both urokinase plasminogen activator (uPA) and tissue plasminogen activator (tPA), PAI-1 inhibits plasma fibrinolysis; this dampening in fibrinolysis makes PAI-1 expression positively associated with cardiovascular disease [38]. However, further research into PAI-1 has revealed that this adipokine is involved in mediating angiogenesis and atherogenesis, but also the development of obesity and insulin resistance [4].

Despite the aforementioned cells representing one source of PAI-1, PAI-1 is also synthesized and released from the stromovascular fraction of WAT; PAI-1 expression is greater in visceral WAT compared to SC WAT [7]. Research into this adipokine has shown that levels of circulating PAI-1 increase with both obesity and states of insulin resistance as found in T2DM. Furthermore, the extent to which PAI-1 correlates with these pathologies is demonstrated through studies showing that PAI-1 could reliably delineate both the risk of developing T2DM and the severity of the metabolic syndrome [39].

Presently, the mechanisms that intertwine both PAI-1 and obesity still require further research. It is believed that the initial increase in this adipokine is attributed, in part, to the concurrent rise in expression of TNF-*α* [40]. As mentioned before, obesity is underpinned by a chronic state of inflammation resulting from the increase in expression of inflammatory mediators such as TNF-*α* and IL-6 [9]. However, the TNF-*α* is believed to act in both an autocrine and an endocrine manner, consequently increasing the expression of PAI-1 [40].

PAI-1 serves as a common denominator in the development of obesity and insulin resistance but also as the casual link to the cardiovascular abnormalities seen in both pathologies [4]. Therefore, direct inhibition of this adipokine has proved to be an attractive area of interest. Known as tiplaxtinin (PAI-039), this oral antagonist of endogenous PAI-1 has been shown to demonstrate a myriad of beneficial effects ranging from antithrombotic properties to attenuating the degree of insulin resistance, adiposity, and body weight [41]. Nevertheless, as it seems to be a recurring theme with the majority of adipokines, translation of such findings to clinical trials involving obese and/or insulin resistant humans has yet to occur. One justification for this involves the disruption to adipocyte differentiation due to PAI-1 antagonists. It is thought that if significant impairment in adipocyte differentiation occurs, this may trigger a toxic accumulation and infiltration of lipids in nonadipose tissue. This process is known as lipodystrophy and is characterized by a combination of pancreatic and renal dysfunction and both skeletal muscle and hepatic steatosis [38]. If this theoretical risk can be nullified, PAI-1 antagonists may prove to be a useful adjunct in the armamentarium against obesity.

## 8. Visfatin

Visfatin, otherwise known as pre-B-cell colony enhancing factor and nicotinamide phosphoribosyltransferase (Nampt), is a 52-kDa cytokine that derives its name due to its expression occurring from adipocytes predominantly found in the visceral WAT [42]. First characterized as an adipokine in 2005, visfatin exhibited a distinct ability to mimic and sensitize the effect of insulin. By binding to insulin receptors, visfatin induces an overall hypoglycemic effect through simultaneous repression of hepatocyte mediated glucose release and augmentation of glucose utilization by peripheral tissues [42]. Molecularly, such effects arise due to the NAMPT activity of visfatin which is the rate-limiting enzyme in the nicotinamide adenine dinucleotide (NAD) pathway underpinning glucose mediated pancreatic *β*-cell secretion of insulin [9]. However, the paper was retracted following failure to replicate their initial findings. Nevertheless, following a meta-analysis determining the significance of visfatin, it was conclusively shown that this adipokine was indeed linked to glucose homeostasis and correlated positively with and was increased in metabolic disorders such as obesity, T2DM, and metabolic syndrome [43].

Although the reason for this rise is not fully understood, it is believed that the increase in circulating visfatin may occur in an attempt to compensate for the insulin resistance and hyperglycemia seen in such pathologies [9]. Therefore, aiming to exploit this phenomenon, researchers have investigated the efficacy of administrating exogenous visfatin. One example of this includes the study by Yoshino et al. [44] which reported a markedly significant improvement in hepatic sensitivity to insulin in addition to altogether ameliorating the state of insulin resistance following administration of visfatin in both obese and T2DM mice. Despite highlighting the efficacious properties of visfatin, its transition to humans may not be so easy due to the positive association between this adipokine and the development of atherogenesis, vascular dysfunction, and inflammation [43].

## 9. Chemerin

If leptin is described as the forefather of adipokines, then chemerin can be deemed the infant of the adipokine family. Only identified as an adipokine in 2007 [45], chemerin had been previously considered to act as a chemoattractant for various cells of the immune system [46]. Although the range of effects orchestrated by this newly identified adipokine have yet to be catalogued, its function varies depending upon the cell in question. This discrepancy is exemplified by the potentiation of insulin signaling at the adipocyte level whilst, in contrast to this, chemerin induces insulin resistance in skeletal muscle cells [9].

Within the obese state, levels of chemerin have been identified as increasing but additionally have been positively correlated with elevations in parameters such as blood pressure and lipid levels [45]. Indeed, following periods of dietary-induced and/or bariatric surgery-related weight loss, circulating levels of chemerin decrease significantly [47]. Despite still being in its infancy, research into this adipokine may reveal startling pharmaceutical therapeutic applications for chemerin relating to obesity and/or obesity-related metabolic disorders.

## 10. Conclusion

2014 marks the 20th anniversary since the identification of leptin. Not only did this monumental occasion identify that adipose tissue was a dynamic and adaptive endocrine hub but also, proved the existence of adipokines. Ranging from the regulation of satiety to glucose homeostasis, adipokines are involved in a plethora of physiological functions [4]. However, as we dissect each member of the adipokine family further, it is apparent that they too are culpable for their involvement in disease. Indeed, in conditions such as obesity and obesity-related metabolic disorders such as insulin resistance, there exists a dysfunctional expression of adipokines [8]. Although additional, undiscovered adipokines may complete the final pieces of the puzzle, our current jigsaw reveals a sophisticated picture which intertwines disease and adipokines. One current mechanism integrating the two is that obesity results in hypertrophy of adipose tissue which in turn induces hypoxia. The resulting hypoxia is said to trigger an inflammatory response, thereby dampening the expression of adiponectin, whilst increasing the secretion of leptin, TNF-*α*, and IL-6. Through the increase in TNF-*α* expression, levels of circulating PAI-1 are said to also be elevated. As to how both visfatin and chemerin integrate within this complex cascade of events, it is still to be discovered [9]. Despite hurdles existing for all of the adipokines mentioned in this review, the most prominent being a lack of definitive human clinical trials, use of these agents may serve as a novel pharmaceutical therapy against rampant epidemics such as obesity. With further research, the potential exists to still yet isolate an adipokine “from fat” that helps us “fight fat.”
